# Dynamics of Dendritic Ice Freezing in Confinement

**DOI:** 10.1021/acs.cgd.1c01488

**Published:** 2022-03-14

**Authors:** James M. Campbell, Bjørnar Sandnes, Eirik G. Flekkøy, Knut Jørgen Måløy

**Affiliations:** † PoreLab, the Njord Center, Department of Physics, University of Oslo, N-0316 Oslo, Norway; ‡ College of Engineering, Swansea University, Crymlyn Burrows, Swansea SA1 8EN, U.K.

## Abstract

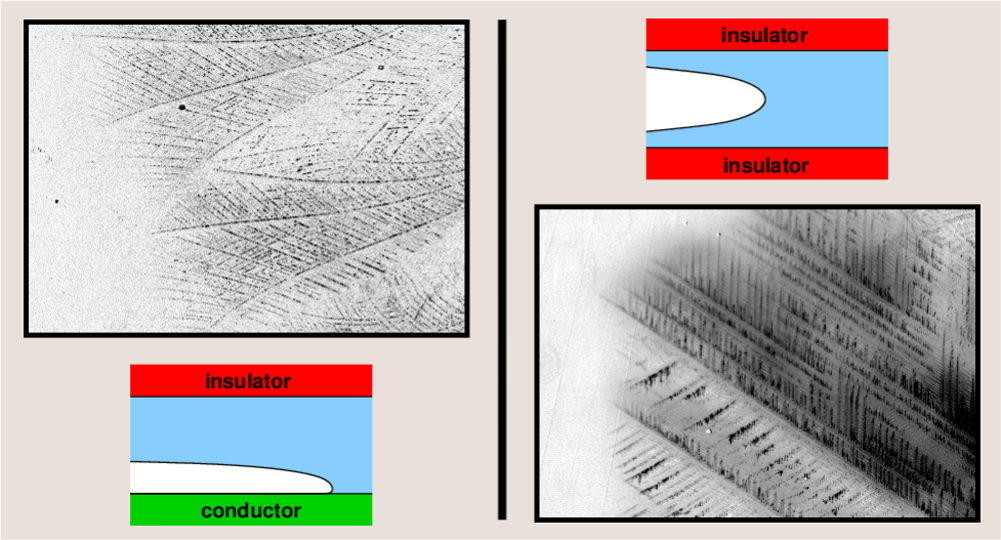

We use high-speed photography to observe the dendritic freezing of ice between two closely spaced parallel plates. Measuring the propagation speeds of dendrites, we investigate whether there is a confinement-induced thermal influence upon the speed beyond that provided by a single surface. Plates of thermally insulating plastic and moderately thermally conductive glass are used alone and in combination, at temperatures between −10.6 and −4.8 °C, with separations between 17 and 135 μm wide. No effect of confinement was detected for propagation on glass surfaces, but a possible slowing of propagation speed was seen between insulating plates. The pattern of dendritic growth was also studied, with a change from curving to straight dendrites being strongly associated with a switch from a glass to a plastic substrate.

## Abstract 2

Ice is frozen between parallel plates to look for a confinement effect upon the speed of dendrite propagation. No firm evidence of such an effect is found with confinements down to 20 μm and temperatures up to −5 °C. We note a strong change in dendrite pattern when moving from a conductive to an insulating substrate.

## Introduction

The dynamics of ice growth in supercooled water are dominated by the dispersion of latent heat. This is because the amount of latent heat produced upon freezing far exceeds the amount that can be absorbed by the heat capacity of the water itself, at any realistic freezing temperature. The consequence of this is that water tends to freeze in two distinct phases. The first is dendritic freezing, whereby thin dendrites of ice grow rapidly, shedding their latent heat into the water in-between. This process can only proceed until the temperature of the interdendrite water reaches the melting point *T*_*m*_. From this point onward the remaining water may only freeze by shedding heat to a reservoir outside the system, typically a process orders of magnitude slower than the dendritic freezing phase.

Ice dendrites grow with a characteristic speed limited by how fast they can exchange heat with their surroundings.^1^ Ivantsov in 1947 found a solution to the heat equation in the form of a parabolic dendrite with constant speed *v* and tip radius *r*,^2^ but this only offered a prediction for the product *vr* as a function of supercooling Δ*T* rather than a unique value for *v*. To predict *v*, a further relationship between *v* and *r* was required, and this was provided in 1978 by Langer and Müller-Krumbhaar,^3^ who found that the Ivantsov solution was unstable above a critical *v*, and who suggested that propagation would occur at this critical speed. This theory predicts experimental observations of dendrite propagation speed very well at low Δ*T* up to about 7 °C.^4^ At higher Δ*T* experimental observations show considerably lower *v* than the theory predicts ;^5^ this is attributed to kinetic limitations of molecular rearrangement becoming another important limitation at higher growth rates.

This body of theory applies to dendrites propagating through bulk water. However, experiments have also shown that very much greater values of *v* may be achieved at a given Δ*T* if the dendrite is allowed to instead grow along a thermally conductive external surface.^6−9^ The effect is attributed to the fact that a dendrite adjacent to a conductive wall may shed its excess latent heat much more easily than may a dendrite in bulk water. Experiments have also shown that the presence of a thermally insulating wall offers no measurable speed increase.^7^

**Figure 1:**
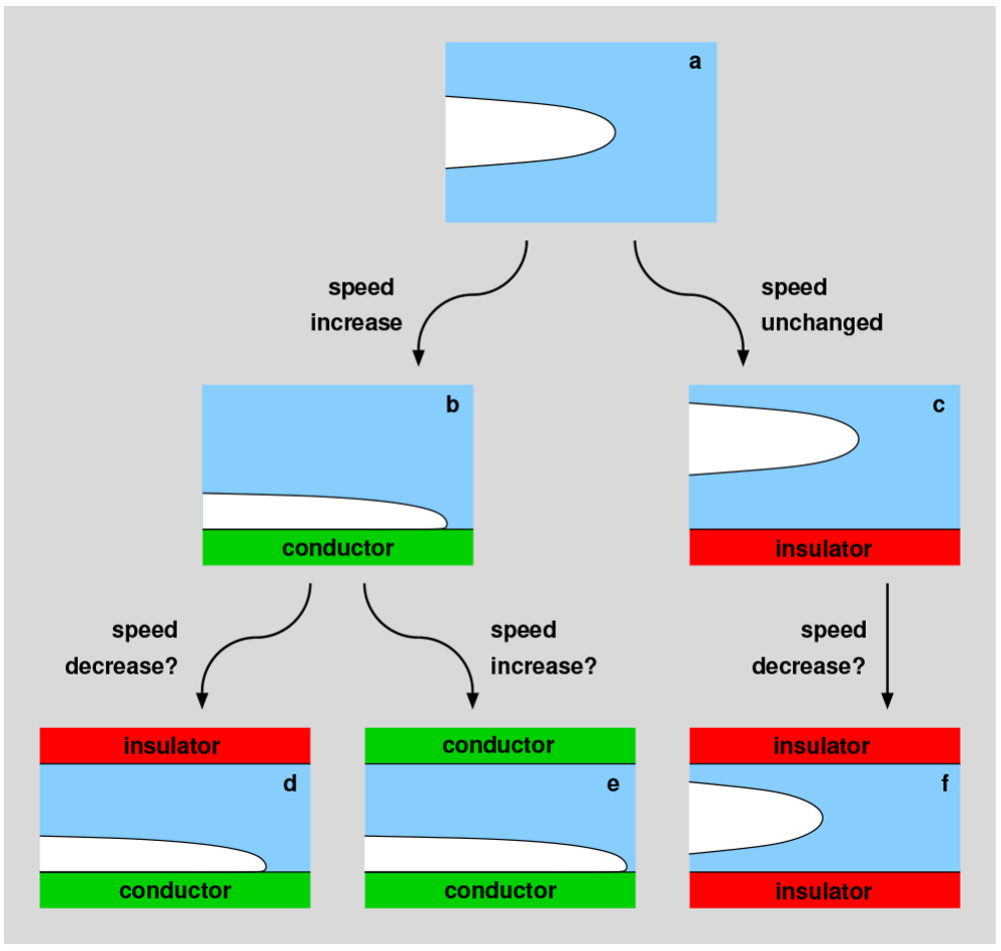
Illustration of the proposed influence of increasing levels of confinement upon ice dendrite propagation speed *v*: (a) bulk propagation; (b) increased *v* in the presence of single conducting substrate; (c) no change in *v* from presence of single insulating substrate (dendrite is not forced to grow near insulator); (d) decrease in *v* (relative to single conductor) from close proximity of insulator; (e) further increase in *v* (relative to single conductor) from second conductor nearby; (f) decrease in *v* relative to bulk propagation as dendrite is forced to grow close to two closely spaced insulators.

One objective of the present work is to determine whether the presence of a confined geometry can compound this thermal influence upon *v*. The concept is illustrated in Figure 1. Consider a dendrite propagating along a thermally conductive substrate, as in Figure 1b. Its latent heat is being dispersed into the substrate, but also into the water on the other side of the dendrite. If a second surface—thermally insulating—were to be placed parallel to the conductive surface with a small spacing between them (Figure 1d), it might be supposed that this second surface will disrupt the dispersion of heat through the water and cause a slight decrease in *v*. This situation is analogous to ice growth in a thin film on the surface of a conductive material. Another situation, shown in Figure 1e, is if a second conductive surface was placed parallel to the first. This could aid heat dispersal and increase *v* ever further, and it is analogous to what could happen within the pore space of a thermally conductive material.

What about an insulating substrate? It might be assumed that a dendrite propagating along an insulating material would not be able to shed its latent heat as easily as it could in bulk and would grow slower; however, in practice, this would never be observed in the case of a single surface since the dendrite would tend to propagate instead further away from the substrate where its retarding influence is not felt (Figure 1c). However, if we have two closely spaced insulating surfaces—analogous to the pore space of an insulating material, or a thin film on an insulating surface—the dendrite has no option but to grow close to the surfaces, and we should expect a reduced *v* in comparison to a free dendrite.

Numerical and analytical studies have addressed the problem of dendritic growth within a two-dimensional perfectly insulating channel, and the solutions they discovered were stable only at extremely high Δ*T*, far above those typical of real-world ice freezing problems.^10−12^

We have tested the scenarios shown in parts d and f of Figure 1 experimentally, measuring dendrite velocities in water with Δ*T* between 4.8 and 10.6 °C, sandwiched between substrates of glass and plastic with separations between 17 and 135 μm. We find no definitive evidence of confinement affecting the propagation speed within this range of parameters, although there is some suggestion of confinement-induced slowing between two insulators.

Another objective of the work is to study the pattern formation of dendritic ice growth along surfaces. Our experimental geometry is naturally suited to this task, as we are confining the growth to a quasi-two-dimensional space amenable to imaging. We find a clear dependence of the pattern type upon substrate thermal conductivity, with dendrites forming mostly straight, angular arrays on insulating substrates and curving patterns on conductive substrates.

## Methods

**Figure 2:**
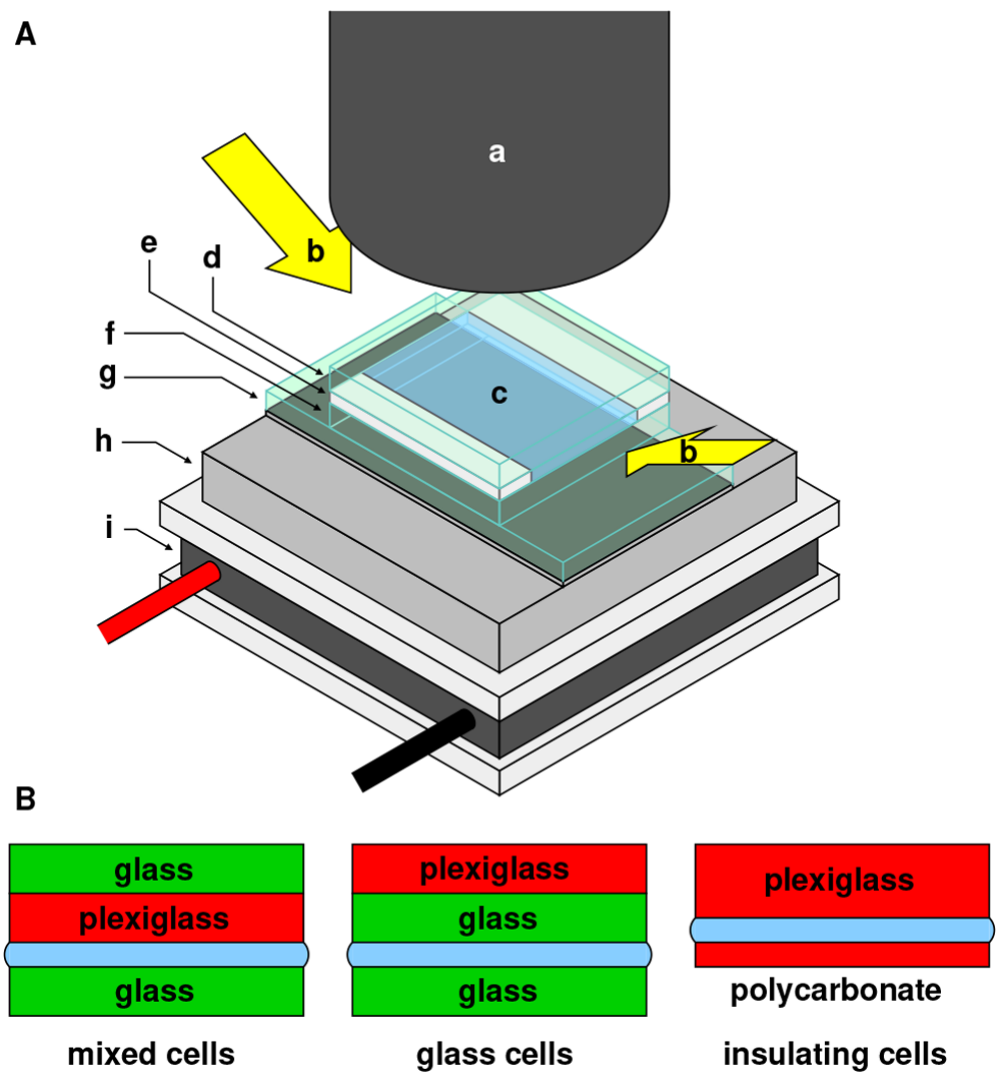
Schematic illustration of the experimental setup (not to scale). (A) Overview, illustrating: (a) macro lens of high-speed camera; (b) incoming light; (c) sandwiched water layer; (d) top plate(s) of cell; (e) plastic spacers; (f) bottom plate of cell; (g) glass mirror; (h) aluminum block with embedded thermocouples; (i) Peltier element. (B) Cross sections of the three types of cells used.

Three types of cells were constructed: mixed cells, glass cells, and insulating cells. All cells were constructed from two parallel plates separated by narrow plastic spacers, with distilled water filling the remaining gap between the plates. Figure 2 shows the experimental apparatus and types of cells.

Mixed cells used a bottom plate of silica glass and a top plate of plexiglass, each sized 25 × 25 × 1 mm. Glass cells used silica glass substrates of the same dimensions for both the top and bottom plates. A plexiglass plate was placed on top of the upper glass plate to insulate the cell from the thermal influence of the warm air above; a glass plate was also placed on top of the upper plexiglass plate of the mixed cells to maintain a constant weight of top plate across both these cell types. Insulating cells were made from asymmetrically sized top and bottom plates in order to maximize the thermal influence of the cooling plate below relative to the room-temperature air above: the bottom plate was 25 × 30 × 0.8 mm polycarbonate, while the top plate was 25 × 30 × 2.9 mm plexiglass. Material thermal properties are listed in Table 1. No properties are listed for polycarbonate due to a shortage of available data for our substrates; instead the values for plexiglass are assumed, these being also typical for the polycarbonate material family.

**Table 1:** Table of Thermal Properties of Materials^a^

material	*c*_*p*_	*k*	κ	ϵ
ice	2.07	2.2	1.2	2.05
water	4.22	0.556	0.132	1.53
silica glass	0.69	1.23	0.81	1.37
plexiglass	1.25	0.18	0.12	0.52

a Heat capacity *c*_*p*_ (J K^–1^ g^–1^), thermal conductivity *k* (W m^–1^ K^–1^), thermal diffusivity κ (mm^2^ s^–1^), and thermal effusivity ϵ (kJ s^–0.5^ m^–2^ K^–1^).^13−18^

Cooling was provided by a Peltier element, upon which was placed an aluminum block in which were embedded eight thermocouples. Temperature measurements were taken from the median of the eight readings. Thermally adhered upon this block was a silvered glass mirror, and upon this was placed the cell. Illumination was provided by two white LED light sources at about 45° off vertical, effectively providing darkfield illumination such that only light scattered from an interface or particle within the cell could be deflected upward into the observing camera. This camera (Photron FASTCAM SA5) was a monochrome 1024 × 1024 high-speed camera recording at between 60 and 500 frames per second, fitted to a macro lens giving a field of view of 4 × 4 mm.

To avoid problems with condensation and frosting on the top surface of the cell, the entire apparatus was contained within plastic tenting and flushed continually with dry nitrogen gas. This was not found to be necessary with the insulating cells, so no gas flow was applied in this case to help reduce temperature gradients through the cell caused by the gas flow.

Crystallization was triggered by cooling the system at 0.35 °C per minute until nucleation spontaneously occurred. The temperature in each experiment was therefore a random product of nucleation rather than a controlled variable. The first run with each cell was solely to generate ice crystals on which the camera could be focused. Two or three experimental runs were then performed, with the crystallization process filmed and the temperature recorded. The temperature was taken to +5 °C between runs to remove ice. After typically the first experimental run, the temperature was taken very slowly above the melting point at 0.05 °C per minute, taking photographs every minute. These photographs were used to establish the temperature at which the ice crystals were judged to begin melting, and any difference between this temperature and 0 °C was added to all recorded temperatures for that cell to account for possible differences between the recorded temperature and the real temperature of the water. The error in temperature is based on the error in determining the melting point, with an additional 0.1 °C error added in quadrature.

Numerous cells of each type were constructed, with a range of different plate separations produced by varying the widths of the plastic spacers. Two different plastic films were used as spacers, with measured thicknesses of approximately 10 and 90 μm. In some cells, two or three layers of the thin sheet were layered to produce intermediate plate separations. However, the values of plate separation reported in this paper are not calculated from these measurements of the spacers. One reason for this is because thin films may sometimes contain trapped bubbles, wrinkles etc. if laid imperfectly, which effectively increase their thickness dramatically by an unknown extent. Also, for smaller spacings it is likely that capillary pressure acts to keep the cells from closing down tightly, since the trapped water extends right to the edge of the cell and the cells are closed only by the weight of the top plate. Instead, the thickness of the water layer is estimated by how long a time it takes to completely freeze.

**Figure 3:**
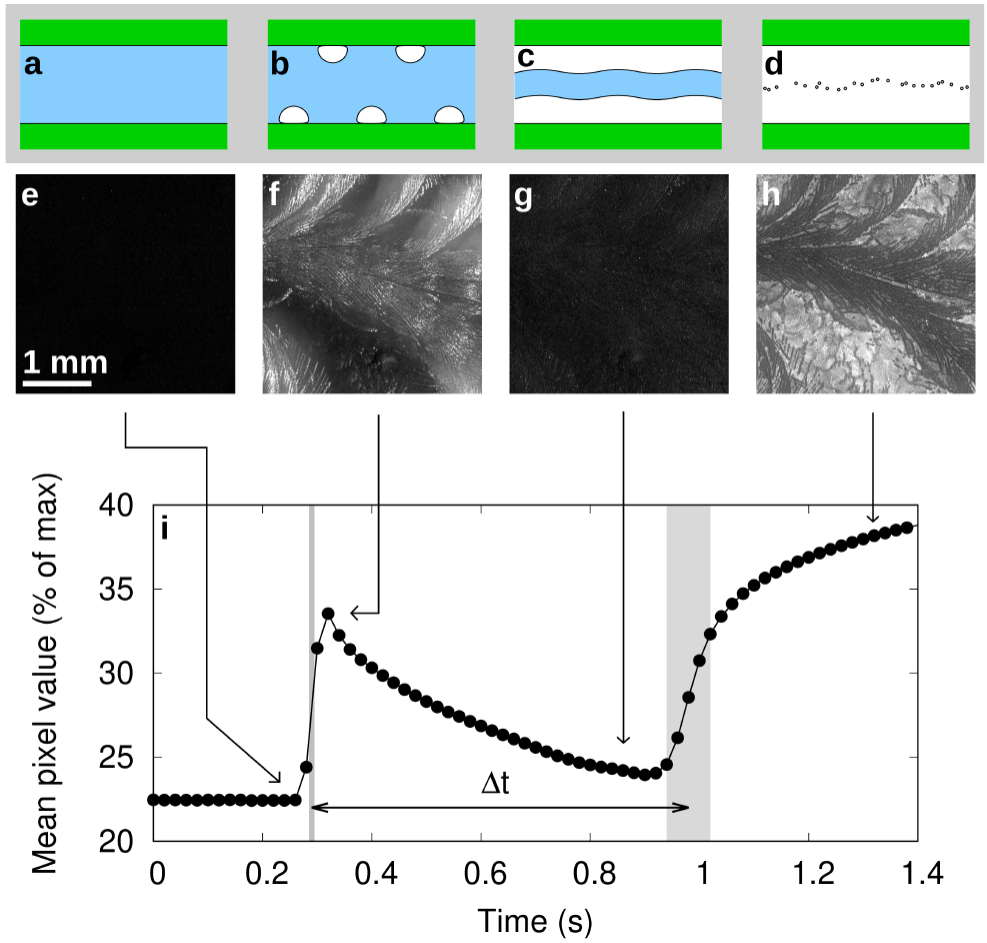
Illustration of the two-stage freezing process. (a–d) Schematic illustration: (a) unfrozen supercooled water; (b) initial dendritic freezing, raising the water temperature to 0 °C and scattering light from the new interfaces; (c) ice growth from either surface through heat diffusion into the substrates, smoothing out the dendrites and reducing the scattering of light; (d) dissolved gases produce many small air bubbles as the last water freezes, strongly scattering light. (e–h) Photographs from an experiment in a glass cell following subtraction of the photograph at *t* = 0 s, corresponding to the stages a–d above. (i) Graph of average pixel value of images in the same experiment (note that only every 10th frame is plotted), showing two sharp increases in intensity corresponding to the first and completion of the second phase of freezing. Arrows indicate the position of the photographs (e–h) in the sequence. The time difference Δ*t* can be used to estimate the plate separation, in this case 98 ± 5 μm. Gray shaded areas indicate the uncertainty in Δ*t*.

Figure 3 illustrates the concept. The period Δ*t* indicates the time delay between the initial, dendritic freezing and completion of the slower freezing of the remaining water between the dendrites. The timing of this completion is very clear from the footage, since the last stage of freezing results in dissolved gases within the last of the liquid water being forced out into small bubbles, producing a dramatic brightening of the image. Since freezing of the interdendrite water is limited by the shedding of its latent heat into the substrates on either side, if we know the thermal properties of these substrates, then we can calculate the plate separation which would result in a freezing time Δ*t*. The mathematics of this is presented in the Appendix. The completion of the second freezing phase is not a sharply defined moment, as seen in Figure 3i, leading to an uncertainty in Δ*t*. This is taken into account within reported uncertainties on calculated plate separations, along with uncertainties in freezing temperature and in material thermal properties.

**Figure 4:**
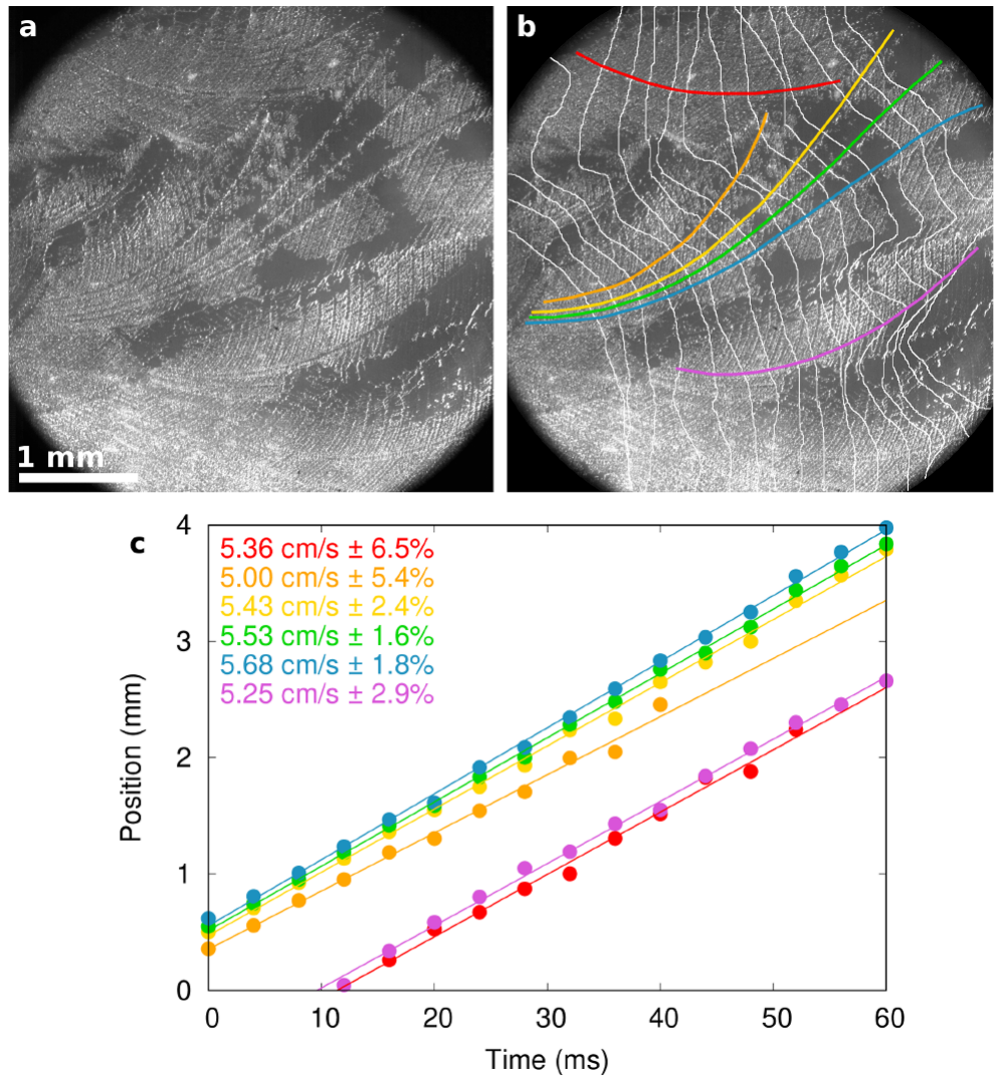
Illustration of dendrite speed measurement technique. (a) Photograph of freezing result in a mixed cell, 33 ± 2 μm plate separation, −6.6 ± 0.2 °C. (b) Same image labeled with positions of freezing front every 4 ms (white lines, estimated from frame-by-frame inspection) and six dendrites selected for study (colored lines). (c) Intersections of dendrites and freezing fronts, measured as distance along each dendrite, for the six dendrites shown above, indexed by color. Lines show a linear fit for each dendrite, whose gradient gives the growth speed (vertical separation of lines is due to arbitrary choice of dendrite start point and is not significant).

Dendrite propagation speeds were measured for a selection of dendrites for each freezing run, as illustrated in Figure 4. The dendrites chosen were those which were felt would give the most precise measurements: they needed to be single continuous dendrites (without kinking onto side branches) and at least 2 mm long and to be clear and unambiguous along their whole length in the footage. The number of qualifying dendrites on each run varied considerably, from as high as 10 in some cases to none at all in many others. The position of the front of the dendrite tips at regular time intervals was manually estimated from frame-by-frame analysis of the high-speed footage. The intersection of these fronts with the dendrites was plotted with distance (following the curve of the dendrite) against time, as in Figure 4. A linear fit then estimated the growth speed and corresponding uncertainty. Dendrites having fewer than six points on this graph were disqualified.

## Results

**Figure 5:**
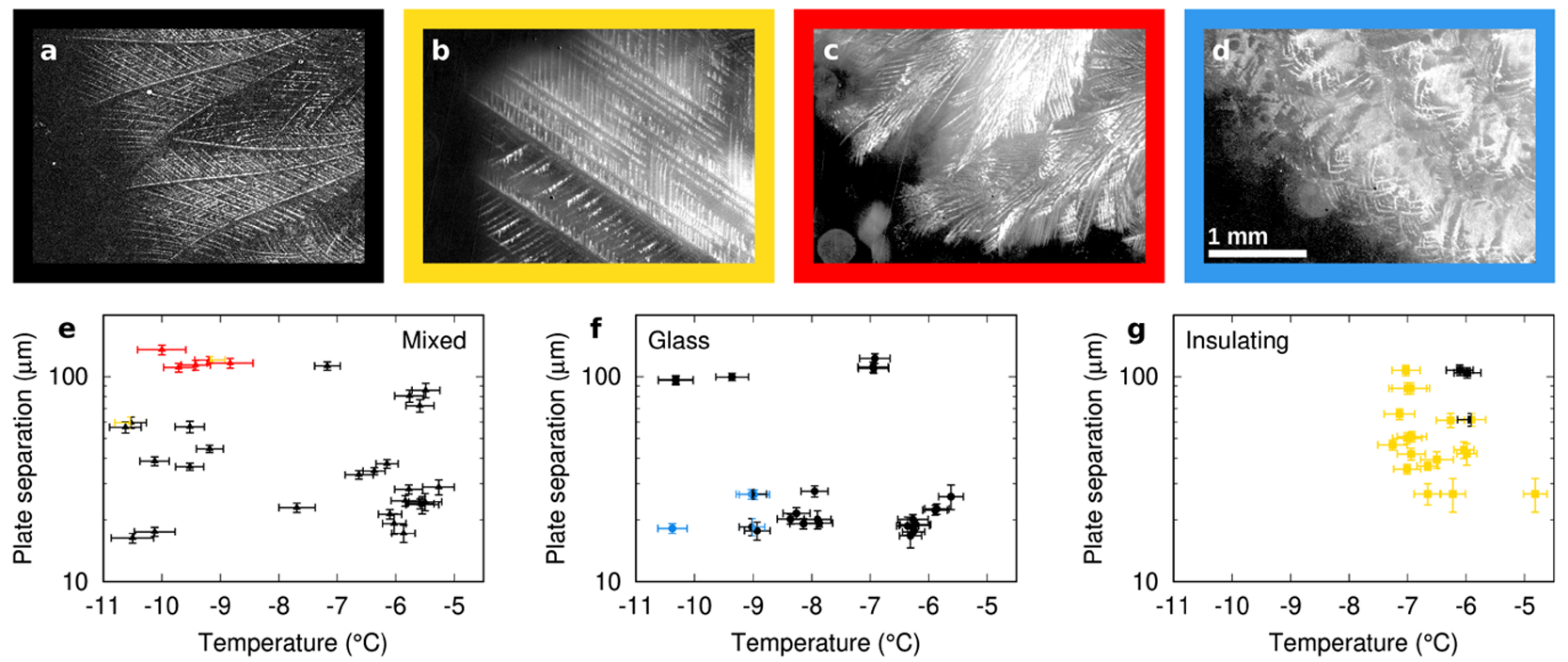
Dependence of dendrite growth pattern upon temperature, confinement and substrate. (a–d) Frames from high-speed footage of the dendritic freezing process (following image subtraction from a frame prior to freezing), illustrating four types of dendrite pattern: (a) stably curving dendrites, two-dimensional; (b) straight dendrites, two-dimensional; (c) stably curving dendrites, three-dimensional; (d) unstably curving dendrites. (e–g) Graphs showing the temperature and calculated cell spacing of every freezing event: (e) mixed, (f) glass, and (g) insulating cells. The color of points represents the pattern of dendrites, corresponding to the borders around images a–d. Where a point is two colors, this represents more than one dendrite morphology within the field of view.

Altogether, 10 mixed cells, nine glass cells, and nine insulating cells were constructed, with which were performed a total of 30, 26, and 20 experimental runs, respectively. Freezing temperatures ranged between −4.8 and −10.6 °C, although temperatures for insulating cells were never lower than −7.3 °C, probably due to frosting on the edges of the cell triggering crystallization before low temperatures could be reached. Plate separations were estimated to range between 17 and 135 μm. This range was fairly consistent across the different cell types and across the range of freezing temperatures despite huge variations in the freezing time Δ*t* from which the plate separations are derived; this consistency lends support to the estimation technique laid out in the Appendix. Figure 5 presents the temperatures and plate separations sampled for each cell type.

Figure 5 also colors the data point for each run according to the pattern of dendrite growth. Four broad categories of growth pattern were identified. The first—and by far the most common in mixed and glass cells—is a two-dimensional pattern of curving dendrites (Figure 5a). “Two-dimensional” in this context means that the dendrites do not have enough room in the third dimension to grow over or under another, meaning that the dendrite pattern viewed from above is a branching tree of nonintersecting dendrites. The second pattern—the dominant one in insulating cells—is also two-dimensional, but here the dendrites are perfectly straight, with their branches at fixed angles (Figure 5b). A third pattern—seen in mixed cells at low temperatures and high plate separations where the cell is very wide compared to the size of the dendrites—is a curving pattern of dendrites similar to the first pattern except with a three-dimensional structure, i.e., dendrites grow over or under other dendrites (Figure 5c). A final pattern—seen only in a few lower temperature runs in glass cells—is another two-dimensional pattern of curving dendrites, distinct from the first. To clearly distinguish the two, we introduce the concept of stable or unstable curvature of dendrites. Stable curvature we define as such that a dendrite growing approximately normal to the growth front tends to remain approximately normal to it, producing long, continuous curving dendrites as seen in Figure 5a. Unstable curvature we define as such that dendrites appear to curve away from the direction normal to the growth front. This has the consequence that dendrites are periodically overtaken by their own side branches, producing a scale-like pattern such as that in Figure 5d.

The straight, stably curved and unstably curved pattern types appeared to be discrete options for dendrite growth, rather than points on a continuum; i.e. there were no intermediate scenarios and no ambiguity as to which pattern best described any specific run. In a few cases, more than one pattern type was seen within a single run, in which case there was a clear boundary between the two types of growth.

It is clear from Figure 5 that the pattern of growth depends strongly on the cell type. In insulating cells, the normal was for straight dendrites, with only a few cases of stably curving dendrites. For mixed and glass cells the normal was stably curving dendrites. However, in glass cells at low temperatures and low plate separations there were a few cases of unstably curving dendrites, and in mixed cells at low temperatures and wider plate separations there were a couple of instances of straight dendrites.

**Figure 6:**
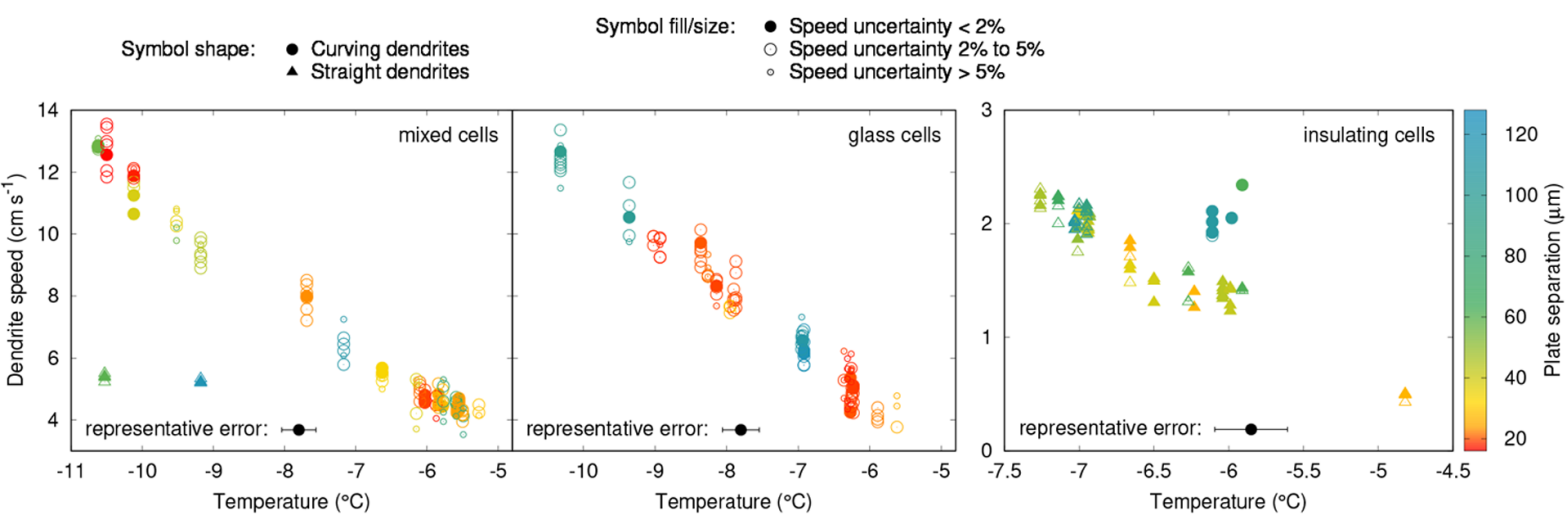
Graphs showing measured dendrite propagation speeds as a function of temperature for each cell type. Symbol color represents plate separation, as quantified by the color bar on the right. Circles represent curving dendrites and triangles straight ones. Filled symbols represent speed measurements with uncertainty below 2%, large open symbols those with uncertainty between 2% and 5%, and small open symbols those with uncertainty greater than 5%. The black point at the bottom of each graph shows the average temperature uncertainty.

Figure 6 shows the results of dendrite propagation speed measurements. Results shown are only for two-dimensional stably curved dendrites and straight dendrites, due to difficulties identifying sufficiently long, unambiguous, continuous single dendrites with the other two pattern types.

**Figure 7:**
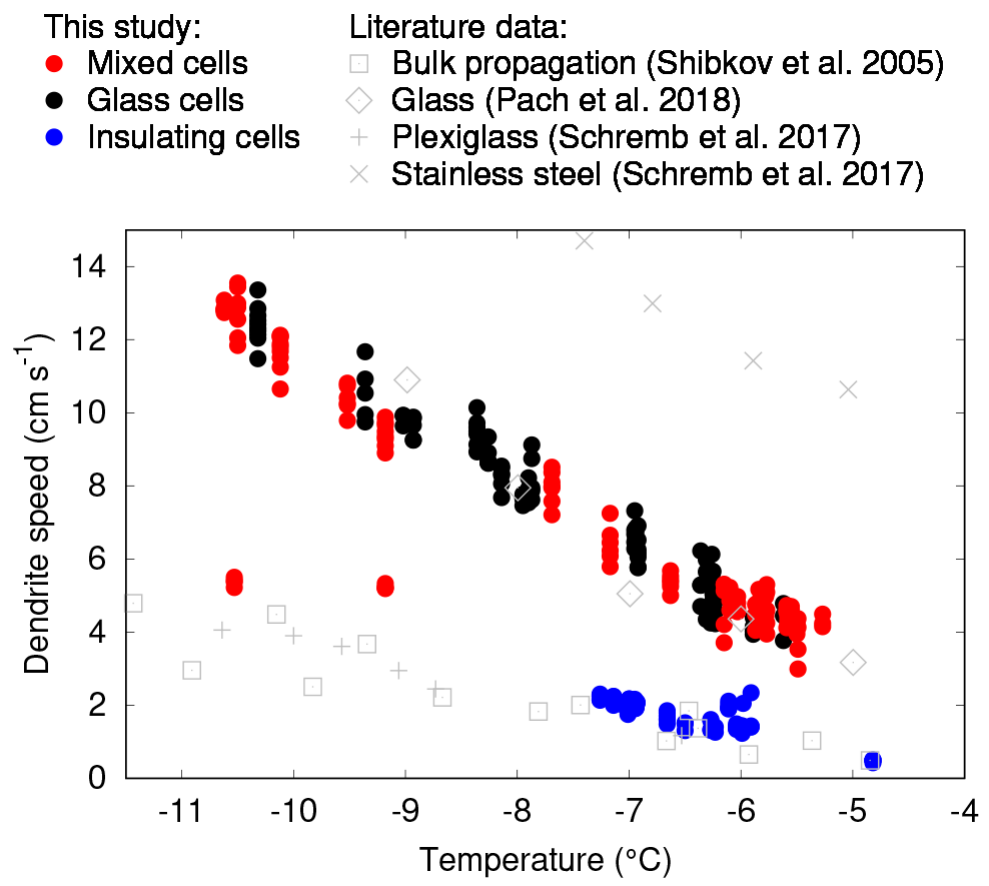
Graph showing measured dendrite propagation speeds in mixed (red), glass (black), and insulating (blue) cells, with literature data for comparison: bulk propagation speeds from Shibkov et al. (squares),^4^ growth speeds on single surfaces of plexiglass (plusses) and stainless steel (crosses) from Schremb et al.,^7^ and glass from Pach et al. (diamonds).^9^

If confinement were having a significant effect upon the propagation speed, we would expect the speed to decrease as mixed cells become narrower at any given temperature, due to the increased proximity of the opposing plexiglass plate to the glass one on which the dendrite is presumed to be propagating. In the glass cells, there would be very little effect, since the opposing glass plate has a very similar thermal effusivity to water. Therefore, we would also expect the propagation speed in narrow mixed cells to be below that in narrow glass cells, a difference which would become less significant as the cells become wider. However, in both the glass and the mixed cells we see no clear dependency of speed upon plate separation and no clear difference between the glass cells and the mixed cells. Speeds are similar to the measured propagation speeds on single glass surfaces in the literature (shown in Figure 7). All of this suggests that we observe growth along a single glass surface, with no noticeable effect from the opposing surface. The only exception to this is the few results in mixed cells where straight dendrites were seen. These propagated at a very much lower speed than did curving dendrites, close to literature results on growth along single plexiglass surfaces. Since both the speed and pattern are suggestive of results in plexiglass cells, and similar results were not seen in glass cells where no plexiglass substrate was present, we suggest that these results may be explained by dendrites propagating along the upper plexiglass surface of the mixed cells, rather than the lower glass one.

For the insulating cells, we would expect narrower cells to exhibit a reduced propagation speed compared to wider ones at the same temperature if the confinement were significant. Looking at the highest temperature freezing events at around −6 °C (the two points above −5 °C are of little practical use with no other plate separations in the same temperature range to compare them to), there is a clear split of the data into two regions based on propagation speed and also dendrite pattern type, with curving dendrites propagating faster than straight ones. This difference is large compared to experimental uncertainties. These faster-moving dendrites also generally had higher plate separations than the slower straight ones, which would be consistent with the higher degree of confinement in the narrower cells slowing growth. However, the correlation is imperfect, and we cannot firmly conclude that plate separation is the underlying factor behind these two styles of dendrite growth.

## Discussion

Figure 5 shows a striking change from straight to curving dendrite patterns as we move from a plexiglass to a glass substrate. In this range of temperatures, similar curving dendrite patterns have been seen in single-surface freezing experiments on glass and other moderately conductive substrates,^6,9^ while straight, angular dendrite patterns have been seen in bulk freezing experiments.^19,20^ Here we show clearly that in the same experimental conditions, a change from one regime to the other may be achieved with a simple change of substrate.

But why does a more thermally conductive substrate produce a transition from straight to curving growth? The straight dendrites seen in insulating cells are certainly a consequence of the hexagonal crystal structure of ice. However, at faster growth speeds, the anisotropy of the crystal faces is likely to diminish in importance relative to the huge temperature gradients driving crystallization. Brener et al. suggested a phase diagram of dendritic growth patterns which features a transition from oriented to isotropic growth with an increase in supercooling.^21^ It may be imagined that the presence of a conductive substrate has a similar effect on dendrites as an increased supercooling, as both increase the efficiency with which latent heat may be removed from the dendrite tip, leading to faster growth rates.

A couple of experiments in mixed cells were seen to exhibit straight growth patterns and much lower growth speeds, possibly a consequence of growth on or near to the plexiglass substrate rather than the glass one. Several experiments in insulating cells also produced a curving pattern, the explanation for which is not known. We do not believe it to be a consequence of confinement, as it was only seen in relatively wide cells. The unstably curving pattern seen in some glass cells we hypothesize to be a consequence of a less common crystal orientation relative to the orientation of growth, and it was not observed sufficiently often to draw conclusions as to the conditions in which this pattern may occur.

No analytical solution has been found to describe the propagation of a dendrite near to a thermally conductive surface, although Brener et al. have described an approximate solution to propagation near to a perfect insulating surface,^22^ and Schremb et al. have put forward a much more empirical model to predict the speed of dendrites along surfaces of varying thermal conductivity.^7^

**Figure 8:**
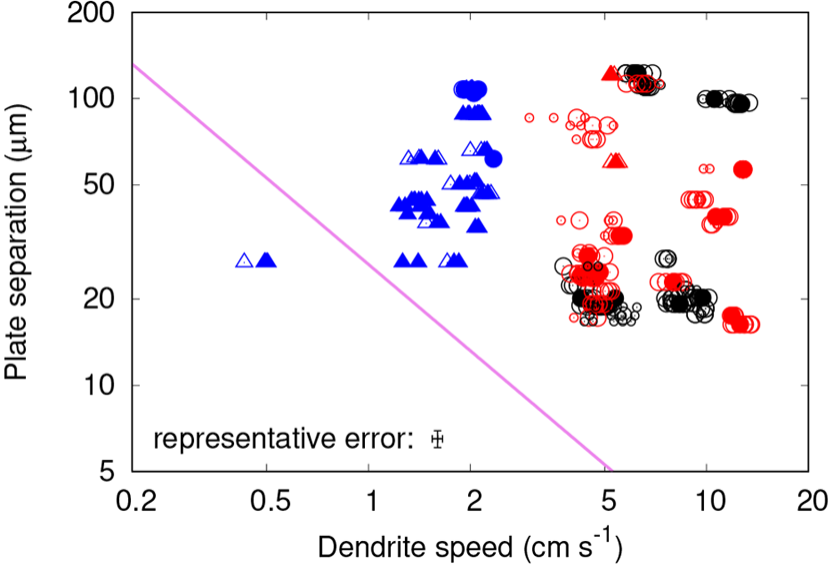
Log–log graph of separation between the two parallel plates vs measured dendrite propagation speeds for all cell types: mixed (red), glass (black) and insulating (blue). Symbol shape, size, and fill follow the same convention as Figure 6. The representative error at the bottom shows average proportional errors across all data. The purple line represents a plate separation equal to the diffusion length of the propagating dendrite. Apart from some experiments in insulating cells, the cells were very wide compared to the diffusion length, explaining the lack of an observed confinement effect upon velocity.

The problem of a dendrite propagating between two substrates of varying thermal conductivity is even more complex than the problem of a single surface, and hence, we have no theoretical model to compare our results against. We can, however, roughly predict the separation of surfaces at which we expect both surfaces to be significant, rather than just one. Models of bulk dendrite propagation define a diffusion length, *d*_0_, to quantify a characteristic length scale of heat diffusion through water in a direction normal to propagation.^3^ This is given by
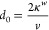
1where κ^*w*^ is the thermal diffusivity of water and *v* the propagation speed. If the plate separation is much larger than *d*_0_ then they are sufficiently far apart that a dendrite may be assumed to propagate along one with little influence from the other, but if the separation is much smaller than *d*_0_ then the thermal influence of both substrates should be of great importance. In Figure 8 we plot the plate spacing of our results against propagation speed, alongside *d*_0_. It may be seen that the large majority of results, including all results in mixed and glass cells, were in conditions of plate spacing much greater than *d*_0_, explaining why no effect of plate spacing was observed. However, some of the results in insulating cells—those at low Δ*T* in narrow cells—were very close to or even below *d*_0_. This lends support to the tentative observation of a confinement effect upon velocity at higher temperatures.

Both dendrite pattern and propagation speed in insulating cells are very similar to those expected for growth through bulk water. We do not know if dendrites tend to propagate through the center of the cell, keeping as far away from the insulating substrates as possible (as illustrated in Figure 1f), or if they propagate along one plexiglass substrate or the other. This probably depends upon whether a dendrite may gain a slight speed boost from a nearby imperfect insulator (in which case we expect it to propagate along the surface), or whether it can only have a negative effect upon the propagation speed (in which case we expect it to propagate as far from the substrates as possible). This has an impact upon the analysis shown in Figure 8, since if the dendrite is propagating through the center of the cell, the relevant quantity to compare to *d*_0_ is half the plate separation, rather than the full plate separation.

One factor which makes it difficult to identify small effects of confinement upon propagation speed is that there is a natural variation in measured speeds between repeat runs in the same conditions and even between different dendrites in the same freezing event, considerably above the uncertainty in our measurement. We understand this to be the consequence of the anisotropy of ice, and varying crystal orientation relative to the direction of propagation. Molho et al. measured the speed of propagation of solidifying crystals within 20 μm glass channels and found the speed to be strongly dependent upon the crystal orientation, although their dendrites were confined to a specific direction rather than merely a specific plane of growth.^23^

## Conclusion

We have studied the dendritic freezing of water between closely spaced parallel plates of varying thermal conductivity. Our primary objective was to look for an increase or decrease in dendrite propagation speed induced by the confinement of the geometry. In the case of mixed and glass cells, no such change in speed was observable, and nothing was seen to distinguish growth within these cells from growth on a single glass surface, with temperatures up to −5.3 °C and plate separations down to 17 μm. Although a negative result, this provides a useful experimental upper bound for conditions in which confinement effects are important to dendritic growth. For insulating cells, the results are less conclusive, as there is some evidence that narrower cells produced lower propagation speeds, but the correlation is not strong enough to definitively reach this conclusion, given the limited number of data points and the significant natural noise between results. We have used the concept of a diffusion length to show how our mixed and glass cells were too wide to expect confinement to be significant within the studied temperature range, but the same argument also shows that many of our insulating cell widths were comparable to the diffusion length.

The other objective of this work—observing the patterns of dendrite growth—was unambiguous in its results. Changing from a conductive glass substrate to an insulating one produces a striking change from curving dendrites to straight, angular ones.

## Appendix

### Estimating Plate Separation from Freezing Time

Consider a flat substrate extending infinitely for all co-ordinates *z* < 0, in contact with water extending infinitely for all co-ordinates *z* > 0. Both are at a uniform temperature *T*^*s*^ = *T*^*w*^ = *T*_*m*_ – Δ*T* (the superscripts *i*, *w* and *s* indicate material properties specific to ice (*i*), water (*w*), or a substrate (*s*)). If the water freezes at time *t* = 0, the initial (dendritic) phase of freezing will raise the temperature of the water throughout to a temperature *T*_*m*_, freezing a fraction *f* of the water into ice, where *f* ≡Δ, the dimensionless supercooling, such that

2

where *L* is the latent heat and *c*_*p*_ the heat capacity, both expressed per unit mass.

Let us assume two things for simplicity: that the initial freezing is instantaneous and that the ice is distributed uniformly throughout the liquid on a sufficiently fine scale to allow us to treat the ice–water mix as a uniform material having a reduced latent heat (1 – *f*)*L*.

Let us also assume that heat conduction is entirely one-dimensional, such that the second phase of freezing will proceed in the form of a growing layer of ice having thickness *h*(*t*). Our goal is to predict the form of this function as a property of Δ*T* and of material properties.

We base our solution to this problem upon solutions to similar problems addressed by Davis.^24^ Within both the substrate and the solid, the heat equation must be obeyed:

3

Here κ is the thermal diffusivity (there is no heat conduction within the liquid, since the liquid is already at *T*_*m*_). Boundary conditions are provided by *T*^*s*^ → *T*_*m*_ – Δ*T* as *z* → – *∞*; *T*^*s*^ = *T*^*i*^ at *z* = 0; *T*^*i*^ = *T*_*m*_ at *z* = *h*(*t*). There must also be a balance of thermal flux across each interface, such that

4

where *k* is thermal conductivity at *z* = 0, and at the growing interface

5

at *z* = *h*(*t*), where ρ is the density.

To solve this, we assume a solution of the form

6

where Λ is a dimensionless parameter to be determined. We can then put the equations into a dimensionless form, reducing *z* and *t* to a single dimensionless variable

7

Note that η^*i*^≢η^*s*^ since κ^*i*^ ≠ κ^*s*^. We can also define a dimensionless temperature, ϑ, such that

8

Now the heat equation may be expressed as

9

with boundary conditions: ϑ^*s*^ → 0 as η^*s*^ → – *∞*; ϑ^*i*^ = ϑ^*s*^ at η^*i*^ = η^*s*^ = 0; ϑ^*i*^ = 1 at η^*i*^ = Λ. Equation 4 becomes

10

at η^*i*^ = η^*s*^ = 0, where ϵ is the thermal effusivity. And eq 5 becomes

11

at η^*i*^ = Λ.

Equation 9 has solutions

12

and

13

within the ice and substrate, respectively. Using the boundary conditions listed above to determine the constants *A*, *B*, *C*, and *D*, we find Λ to be the solution of the equation

14

Given a known Λ(Δ*T*) and a measured time scale Δ*t*, plate separation *w* may be estimated from eq 6, given that *w* = 2*h*(Δ*t*); i.e.. ice is assumed to grow inwards from both substrates. In the case of two dissimilar substrates, two distinct functions Λ_1_(Δ*T*) and Λ_2_(Δ*T*) must be considered, leading to two corresponding functions *h*_1_(*t*) and *h*_2_(*t*). *w* in this case is then *h*_1_(Δ*t*) + *h*_2_(Δ*t*).
